# Reevaluation of the Complete Genome Sequence of Magnetospirillum gryphiswaldense MSR-1 with Single-Molecule Real-Time Sequencing Data

**DOI:** 10.1128/genomeA.00309-18

**Published:** 2018-04-26

**Authors:** René Uebe, Dirk Schüler, Christian Jogler, Sandra Wiegand

**Affiliations:** a Department of Microbiology, University of Bayreuth, Bayreuth, Germany; b Department of Microbiology, Radboud Universiteit Nijmegen, Nijmegen, The Netherlands

## Abstract

Magnetospirillum gryphiswaldense is a key organism for understanding magnetosome formation and magnetotaxis. As earlier studies suggested a high genomic plasticity, we (re)sequenced the type strain MSR-1 and the laboratory strain R3/S1. Both sequences differ by only 11 point mutations, but organization of the magnetosome island deviates from that of previous genome sequences.

## GENOME ANNOUNCEMENT

Magnetospirillum gryphiswaldense MSR-1 was isolated from river sediments in 1990 (1) and classified as the type species of the genus Magnetospirillum (2). It serves as a model organism for the analysis of bacterial magnetotaxis and magnetosome formation (3). A first version of the complete genome sequence, based on Illumina Solexa and Roche 454 reads, was published in 2014 (4). Notably, extensive rearrangements between this genome sequence and a previous draft genome sequence were observed, indicating a high genomic flexibility and “domestication” effects (4). To more deeply investigate this finding with state-of-the-art long-read sequencing, we sequenced one archetypal strain of M. gryphiswaldense, MSR-1, that was directly obtained from the DSMZ strain collection and the often-passaged, spontaneously rifampicin- and streptomycin-resistant lab strain R3/S1 (5).

DNA was extracted and purified using 20/G Genomic-tips (Qiagen, Venlo, The Netherlands). The 10-kb SMRTbell template libraries were prepared according to Pacific Biosciences (Menlo Park, CA) instructions. Briefly, 8 µg of genomic DNA was sheared using g-tubes (Covaris, Woburn, MA). Fragments were end repaired and ligated overnight to hairpin adapters using the DNA/polymerase binding kit 2.0 (Pacific Biosciences). The 4-kb size selection was performed using a BluePippin system (Sage Science, Beverly, MA) according to the supplier’s instructions. Single-molecule real-time (SMRT) long-read sequencing was carried out utilizing P6 chemistry on a PacBio RS II instrument, taking 240-min movies for each SMRT cell.

The 550-bp paired-end short-read libraries were prepared with the TruSeq DNA PCR-free LT library preparation kit (Illumina, San Diego, CA), according to instructions given in the TruSeq DNA PCR-free library prep reference guide's low sample (LS) protocol. The libraries were sequenced on an Illumina MiSeq system, employing a MiSeq reagent kit v3 (Illumina) and 600 cycles.

Long-read genome assembly was performed using the RS_HGAP_Assembly.3 protocol SMRT Portal version 2.3.0 with standard parameters. Final contigs were error corrected by Illumina read mapping and subsequent variant and consensus calling using the Burrows-Wheeler Aligner (BWA), VarScan v2.3.7, and GenomeAnalysisTK (6–8). Resulting consensus sequences were trimmed, circularized, and aligned. Genome annotation was performed with Prokka v1.11 (9).

Both genomes consist of one circularized contig each, having a size of 4,155,740 bp, a G+C content of 63.2%, and 3,980 genes. Unexpectedly, only 11 point mutations could be observed. Two are located within genes described to mediate rifampicin (*rpoB*) or streptomycin (*rpsL*) resistance upon modification (10, 11). These results explain the antibiotic resistances of strain R3/S1 and verify the different sources of the strains. In contrast to previous observations (12), no major genomic rearrangements could be detected. One possible explanation might be the high density of transposases found in the genome (88 genes annotated as transposase/integrase), impeding previous attempts at assembly without long-read data. The significantly reduced size of the new genome sequences also supports this interpretation (4, 13). Alternatively, the previously reported large deletions might occur only under specific stress conditions. Additionally, an ∼10-kb genomic region containing several magnetosome-related genes (e.g., *feoAB1* and *mmxF*) and flanked by transposases, which was absent from a previous draft genome sequence (12) or was located at the 3′ end of the magnetosome island in the first complete genome sequence of M. gryphiswaldense (3), is located at the 5′ region of the magnetosome island in our new genome sequences.

### Accession number(s).

The whole-genome sequences described here have been deposited in GenBank under the accession numbers CP027526 and CP027527.
